# Current Status and Future Directions of Pain-Related Outcome Measures for Post-Surgical Pain Trials

**DOI:** 10.1080/24740527.2019.1583044

**Published:** 2019-07-30

**Authors:** Ian Gilron, Henrik Kehlet, Esther Pogatzki-Zahn

**Affiliations:** a Department of Anesthesiology & Perioperative Medicine, Queen’s University, Kingston, Ontario, Canada; b Department of Biomedical & Molecular Sciences, Queen’s University, Kingston, Ontario, Canada; c Centre for Neuroscience Studies, Queen’s University, Kingston, Ontario, Canada; d Department of Anesthesiology & Perioperative Medicine, Kingston Health Sciences Centre, Kingston, Ontario, Canada; e Section for Surgical Pathophysiology, Rigshospitalet, Copenhagen, Denmark; f Department of Anesthesiology, Intensive Care and Pain Medicine, University Hospital, Muenster, Germany

**Keywords:** Acute pain, chronic pain, analgesia, outcome measurement, postoperative, postsurgical, surgery

## Abstract

**Background**: Clinical trials remain vital in order to: A) develop new treatment interventions, and also, B) to guide optimal use of current interventions for the treatment and prevention of acute and chronic postsurgical pain. Measures of pain (e.g. intensity and relief) and opioid use have been validated for the settings of postsurgical pain and continue to effectively guide research in this field..

**Methods**: This narrative review considers needs for innovation in postsurgical pain trial outcomes assessment.

**Results**: Future improvements are needed and include: A) more widespread measurement of movement-evoked pain with validation of various procedure-relevant movemen-tevoked pain maneuvers; B) new validated analytical approaches to integrate early postoperative pain scores with opioid use; and, C) closer attention to the measurement of postoperative opioid use after hospital discharge. In addition to these traditional measures, consideration is being given to the use of new pain-relevant outcome domains that include: 1) other symptoms (e.g. nausea and vomiting), 2) recovery of physiological function (e.g. respiratory, gastrointestinal, genitourinary and musculoskeletal), 3) emotional function (e.g. depression, anxiety) and, 4) development of chronic postsurgical pain. Also, there is a need to develop pain-related domains and measures for evaluating both acute and chronic post-operative pain. Finally, evidence suggests that further needs for improvements in safety assessment and reporting in postsurgical pain trials is needed, e.g. by using an agreed upon, standardized collection of outcomes that will be reported as a minimum in all postsurgical pain trials.

**Conclusions**: These proposed advances in outcome measurement methodology are expected to improve the success by which postsurgical pain trials guide improvements in clinical care and patient outcomes.

## RÉSUMÉ

**Contexte**: Les essais cliniques demeurent essentiels pour a) développer de nouvelles interventions de traitement et b) guider l’utilisation optimale des interventions actuelles pour le traitement et la prévention de la douleur postopératoire aigue et chronique. Les mesures de la douleur (ex.: intensité et soulagement) et de l’usage d’opioïdes ont été validées pour les contextes de la douleur postopératoire et continuent de guider la recherche dans le domaine.

**Méthodes**: Cet examen narratif porte sur la nécessité d’innover en ce qui concerne les résultats des études sur l’évaluation de la douleur postopératoire.

**Résultats**: Des améliorations sont nécessaires, parmi lesquelles on compte: a) une plus grande généralisation de la mesure de la douleur évoquée par le mouvement avec validation de diverses manœuvres relatives à la douleur évoquée par le mouvement pertinentes aux interventions; b) de nouvelles approches analytiques validées afin d’intégrer les scores obtenus pour la douleur postopératoire précoce et l’usage d’opioïdes; c) une attention plus soutenue à la mesure de l’usage postopératoire d’opioïdes après le congé de l’hôpital. En plus de ces mesures traditionnelles, l’utilisation de nouveaux domaines de résultats pertinents à la douleur sont aussi envisagés, dont : 1) d’autres symptômes (ex. : nausée et vomissements), 2) le rétablissement de la fonction physiologique (ex. : respiratoire, gastro-intestinale, génito-urinaire et musculo-squelettique, 3) la fonction émotionnelle (ex. : dépression anxiété) et 4) le développement de douleur postopératoire chronique. De plus, des domaines et mesures de la douleur sont nécessaires pour évaluer la douleur aigue et la douleur postopératoire chronique. Finalement, les données probantes suggèrent que des améliorations sont nécessaires dans l’évaluation de la sécurité, en utilisant par exemple un ensemble minimal de résultats convenus et standardisés qui seront utilisésdans toutes les études portant sur la douleur postopératoire.

**Conclusions**: On s’attend à ce que les améliorations proposées dans la méthodologie de mesure des résultats permettent aux études sur la douleur de guider les améliorations en matière de soins cliniques et de résultats des patients de manière plus fructueuse.

## Introduction

Over 300 million surgical procedures are done in the world each year^1^ and approximately 60–70% of these are associated with moderate or severe postsurgical pain.^2–4^ The clinical goals for acute postsurgical pain management include the relief of pain-related suffering, and the reduction of pain-related physiological impairment (e.g. immobility, impaired cardio-respiratory, gastrointestinal and cognitive function, disturbed sleep) in order to accelerate functional recovery after surgery.^5,6^ In addition to reducing the severity and adverse effects of postsurgical pain in the days to weeks after surgery, development of chronic postsurgical pain that persists beyond 3 months after surgery has been increasingly recognized as a serious complication that requires greater attention by surgeons, anesthesiologists and other perioperative healthcare providers and researchers.^7–10^

The evolution of clinical trials of postsurgical pain treatment and prevention has demonstrated a dichotomy between: 1) trial designs that facilitate development of novel analgesic agents, for example by the pharmaceutical industry, and, 2) more pragmatically oriented trials focused on guiding improvements in postsurgical clinical outcomes.^11^ Proof-of-concept study designs in trials evaluating novel therapies for postsurgical pain – typically in comparison with inert placebo – generally emphasize trial feasibility, single-dose trial designs, high assay sensitivity (i.e. ability of the trial to demonstrate a treatment versus placebo difference if one exists) and internal validity.^12–14^ In such trials – that typically evaluate novel analgesics that have shown efficacy in reducing nociception in preclinical animal models – primary outcome measurement is usually focused on pain intensity or relief.^14^ Pragmatically oriented study designs of trials focused on optimizing clinical outcomes – often involving currently available pain treatments – generally emphasize generalizability, clinical relevance and importance, and often involve multi-dose trial designs.^11,15^ In such trials, that typically involve various domains of clinical assessment, outcome measures in addition to pain should whenever possible be included, addressing domains such as physiological function, patient satisfaction and time to hospital discharge readiness.^11^ This article discusses the current status and future directions of outcome measures for post-surgical pain trials with a distinction between trials of acute postsurgical pain treatment and chronic postsurgical pain prevention.

## Traditional Pain-Related Outcome Measures in Postsurgical Pain Trials

### Pain Intensity and Pain Relief

Given their focus, clinical trials of postsurgical pain almost always include outcome measures of pain intensity or relief, and most often, one of these measures is defined as the primary outcome of the trial.^16^ In a review of 3 meta-analyses of 154, mostly single-dose, trials of aspirin, acetaminophen and ibuprofen, the most commonly used outcomes were pain intensity, pain relief, global improvement measures, rescue analgesic use, and adverse effects.^17^ Of note, the majority of these RCTs reported trial treatment group averages rather than proportions of responders with a predefined level of treatment response (e.g. 50% pain reduction). This has been recognized to be problematic because very few patients “behave like the average” and a more clinically relevant treatment group measure is the proportion of patients who responded (e.g. received a 50% pain reduction) to the treatment.^18^ Historically, various different rating methods have been used to assess postoperative pain intensity and relief.^14,19,20^ Commonly used rating scales include verbal/category scales (e.g. none, mild, moderate or severe for intensity; none, slight, moderate or complete for relief), numerical rating scales (e.g. 0–10 scale) or visual analog scale (e.g. 0–100 mm scale).^21,22^

Research interest in treatment and prevention of chronic postsurgical pain has been growing^7,23^ given its major negative impact on long-term quality of life.^24,25^ Because chronic postsurgical pain is generally defined as new pain that develops after a surgical procedure (or, in some situations, increased pain intensity after the surgical procedure if a patient presented pain in the operated area before surgery) and persists at least 3 months after surgery,^7,26,27^ some measure of pain at, and/or beyond, 3 months after surgery is typically involved in studies of persistent post-surgical pain. For example, in a meta-analysis of clinical trials of drugs studied for the prevention of chronic pain after surgery, the most commonly used outcomes were *any* (i.e. ≥ zero intensity) pain reported ≥ 3 months after surgery, and, when persistent pain intensity was actually rated, ≥ moderate pain ≥ 3 months after surgery.^28^ It should be noted, however, that more specific definitions and assessment methods of outcome measures may have an important impact on study results, e.g. a single telephone pain intensity rating versus a more robust multiple-day pain diary and also multiple time points beyond 3 months.

### Rescue Opioid Consumption

Over half a century ago, pioneering investigations by Henry Beecher and others actually used postoperative opioid consumption as a measure of pain severity after different surgical procedures – at a time when very few, if any, non-opioid analgesics were available.^29^ With the subsequent investigation into postoperative analgesic efficacy of non-steroidal anti-inflammatory drugs, the use of opioids for analgesic rescue stimulated the need for new analytical approaches to censor and/or impute pain scores for patients after receiving rescue analgesia in a trial.^14,18,30^ The development of patient-controlled analgesia (PCA) systems with data capture^31,32^ provided the ability to assess apparent analgesic responses to other analgesic treatments, as indicated by a reduction in PCA opioid consumption.^33^ Thus, the concept of opioid sparing as an effect of an investigational analgesic led to opioid consumption as an important outcome measure in postsurgical pain trials involving rescue analgesic with non-study opioids.^11,34^

## Future Considerations for Other Outcome Measures

### Needs for Improvement in Postsurgical Pain Trials

Movement-evoked pain as a critically important outcome measure

For several decades, patients, clinicians and researchers alike have recognized the important distinction between pain at rest (PAR) and pain evoked or aggravated by movement (MEP – movement-evoked pain) such that MEP is generally more severe than PAR.^35–37^ Previous studies have suggested an association between MEP and impaired recovery of postoperative physiological function^38^-^40^ and that analgesic interventions with effective reduction of MEP result in improved pulmonary outcomes.^41,42^ Despite this compelling rationale, a previous systematic review of over 1,800 analgesic clinical trials involving thoracotomy, hysterectomy and knee arthroplasty demonstrated that only 39% of trials actually included MEP as a trial outcome and more than half of these trials failed to distinguish between PAR and MEP when defining the trial’s primary outcome.^43^ Despite prominent recognition of this important distinction,^44^ clinical trials continue to fail to distinguish between PAR and MEP when defining their outcomes.^45,46^ Thus, an important future direction for postsurgical trials is to greatly heighten awareness and implementation of MEP, in addition to PAR, as a critically important outcome measure. This must be done in a procedure-specific set-up with assessment of procedure-specific, clinically-relevant movement. Ideal would be the use of agreed upon, reliable and validated patient-reported outcome measure for MEP based on a procedure-relevant “movement” but validation of such measures are needed.

Opioid consumption – an increasingly important outcome measure

As mentioned above, reduced opioid requirements, or opioid sparing, after surgery may reflect the analgesic efficacy of an investigational pain treatment making opioid consumption a popular outcome measure in analgesic trials. However, careful reflection has highlighted several limitations of opioid consumption as an outcome, particularly in the setting of PCA opioid use. In particular, a weak correlation between pain intensity and opioid consumption has been observed, which could be, in part, due to: 1) acute tolerance to opioids, 2) variable training of patients on how to use PCA, 3) other opioid side effects (e.g. nausea-induced reductions in PCA use), and, 4) effects of investigational analgesic on PCA (e.g. sedation-induced reductions in PCA demand).^47^ Thus, interpretation of pain scores, as the sole measure of analgesic treatment response, in postsurgical placebo-controlled analgesic trials is complicated by PCA opioid use because measured efficacy differences between placebo and investigational analgesic can be partially reduced if greater opioid use, and opioid-related analgesia, in the placebo group reduces the placebo versus treatment difference. Also, as discussed above, reliance on opioid consumption data alone also has its limitations in making any conclusions about investigational drug efficacy. For these reasons, investigators have proposed different analytical approaches to integrate pain scores *and* opioid consumption in order to provide a more representative composite score in postsurgical analgesic trials.^48–50^ Since uniform agreement has not yet been reached on an optimal approach, additional research has been recommended to more confidently conclude whether such complex analytical approaches provide a more definitive characterization of the efficacy of an investigational analgesic.^14^

The greatest relevance of postoperative opioid consumption as a clinical outcome is related to the well-recognized adverse effects of opioids affecting neurological, respiratory, gastroenterological and genitourinary systems.^51^ Thus, regardless of the mechanism, if an investigational treatment can effectively reduce opioid use to a degree that will result in reduced opioid-related adverse effects (without increasing pain), this would certainly be a clinically important favourable effect.^52^ In fact, several examples have been described where multiple trials of interventions with even modest analgesic efficacy result in significant reductions in opioid-related adverse effects. For example, postoperative administration of non-steroidal anti-inflammatory drugs reduced nausea and vomiting,^53^ and postoperative infusions of intravenous lidocaine accelerated recovery of postoperative bowel function.^54^ In fact, a specific composite scale designed to assess postoperative opioid-related side effects^55^ has been introduced to demonstrate the impact of novel analgesic interventions to reduce opioid-related side effect burden^56^ and should be used much more in future trials.

More recently, the largely North American opioid crisis has focused more attention on the amount of opioid use before and after surgery.^57^ Although preoperative opioid use and preoperative pain – at or remote from the surgical site – have been shown to be associated with a higher risk of persistent postoperative pain,^58^ some large cohort studies have reported varying, but concerning, rates of chronic postoperative opioid use after minor surgery in previously opioid-naïve patients.^57,59^ As this research continues, it has been recognized that efforts to reduce opioid use, particularly after hospital discharge, requires further attention^60^ and, thus, opioid consumption beyond the early postoperative period is an increasingly important trial outcome measure.

### Do We Need More Outcome Measures?

Recently proposed future research needs for postsurgical pain management include: 1) development of optimal strategies for the management of pain in challenging populations (e.g. preexisting chronic pain, mental health conditions, and substance use disorders); 2) prevention of transition to chronic postsurgical pain; and, 3) post-hospital discharge management of postsurgical pain in home/community settings.^11^ These newer areas of postsurgical pain research may require development and implementation of new patient-centered outcome measures. Such measures will need to be focused on the population of interest (e.g. outcomes related to depression, chronic pain, substance use disorder), or on the surgical procedure of interest (e.g. measures of functional recovery after total knee arthroplasty), or on the intervention of interest (e.g. feasibility outcomes of outpatient indwelling catheters for regional analgesia).

Relevant to the management of early postoperative pain, the Standardised Endpoints in Perioperative Medicine (StEP) initiative used a Delphi approach to develop and propose 6 defined outcomes for the domain of “patient comfort” in perioperative medicine including: 1) pain intensity (at rest and during movement, 24 hours postop), 2) nausea and vomiting, 3) quality-of-recovery, 4) time to gastrointestinal recovery, 5) time to mobilisation, and 6) sleep quality.^61^ However, due to a broader perspective, procedure specific pain-related aspects were not considered and patient’s perspective was not taken into account. Future research is needed to elucidate these further in order to identify (a set of) pain-related outcome domains and instruments that are best suited for certain surgical procedures based on evidence. Important here is a consensus procedure that includes patients, for example similar to an approach recommended by the comet initiative (http://www.comet-initiative.org). Important for measurement instruments to be recommended at the end of clinical trials is feasibility, good content validity and good internal consistency (http://www.comet-initiative.org). In addition, future pain trials should include detailed information on patient-specific pain risk factors, such as pain catastrophizing, pre-operative opioid use or other types of “pain-sensitized” patients.^11^

The setting of chronic postsurgical pain is perhaps more complex and involves potential interventions, and modulating factors, from before the surgical procedure to the early perioperative period to later time points after surgery.^8,9,27^ As such, a broader range of measures have been proposed for research to better understand the development of chronic postsurgical pain and its prevention.^62^ Such measures are associated with the domains of patient demographics, pain-related factors, other clinical diagnoses and comorbidities, surgery-related factors, psychological/psychiatric factors, physical functioning, and, global measures of outcome.^62^ Other domains beyond pain intensity should be considered for exploring effectiveness of preventive intervention and/or treatment of chronic postoperative pain. Ideal would be the use of agreed upon pain-related outcome domains that are similar to those considered for chronic pain in general,^63^ but tailored more specifically to chronic postsurgical pain and tied to a core set of validated instruments.^64,65^ Such an approach would enable the identification of clinically relevant effects of a treatment, direct comparison of the effect of interventions between different trials and aligned evidence-based recommendations.

### Safety Outcomes in Postsurgical Pain Trials

Recognition of the importance of appropriately assessing and reporting safety outcomes in clinical trials has led to publication of the 2004 extension to the CONSORT statement (Consolidated Standards of Reporting Trials) using a structured checklist approach for submission of clinical trial manuscripts.^66^ Despite the publication of this statement, recent reports have suggested that clinical trials published from 2000 to 2011 in the European Journal of Pain, Journal of Pain and Pain failed to meet 4 of the 10 recommendations of the 2004 CONSORT harms extension^67^ and that 40% of such publications failed to report any information regarding serious adverse events.^68^ This area of deficiency has been well recognized in the setting of postsurgical pain trials dating back to the late 1990s.^69^ However, despite the 2004 CONSORT harms extension and the recent reviews suggesting deficiencies in pain trials in general, more recent evidence suggests that safety assessment and reporting in postsurgical pain trials continues to lag behind.^70,71^ Thus, further attention to improving the assessment and reporting of safety outcomes in postsurgical pain trial requires a concerted, collaborative commitment among research investigators, ethics review boards, research funding agencies and journal editorial boards.

## Conclusion

Outcome measures of pain (intensity and relief) remain clinically important and are commonly used in postsurgical pain trials. Given that opioids remain commonly used as rescue medications in trials of early postsurgical pain, measures of opioid consumption continue to be necessary and further research is needed to validate analytical approaches that integrate opioid consumption and pain in efforts to better estimate analgesic efficacy of an investigational treatment. Future initiatives and projects should, however, focus on identifying a consented set of pain-related outcome measures that are clinically relevant (including relevance to patients), evidence-based, sensitive to changes, and validated for its specific use in postoperative patients.^72,73^ A consensus on such a core set of patient-centered outcome measures may be of great value in novel trials involving specific surgical procedures, patient populations and unique analgesic interventions. Finally, assessment and reporting of safety outcomes is critical in postoperative analgesic trials and requires further improvement.
